# Open‐Label, Prospective Study of a Prebiotic Gel Cream on Its Efficacy of Mild to Moderate Acne Management and Effects on the Functional Skin Microbiome

**DOI:** 10.1111/jocd.70138

**Published:** 2025-10-16

**Authors:** Laila Afzal, Ajay S. Dulai, Zill‐e‐huma Khan, Nhi Nguyen, Nasima Afzal, Hemali B. Gunt, Raja K. Sivamani

**Affiliations:** ^1^ Integrative Skin Science and Research Sacramento California USA; ^2^ Burt's Bees Durham North Carolina USA; ^3^ Pacific Skin Institute Sacramento California USA; ^4^ Department of Dermatology University of California‐Davis Sacramento California USA; ^5^ College of Medicine California Northstate University Elk Grove California USA

**Keywords:** acne vulgaris, *Cutibacterium acnes*, microbiome, prebiotics

## Abstract

**Background:**

Acne vulgaris is a chronic inflammatory condition, which is estimated to affect greater than 85% of the population. Acne is a multifactorial condition, which can be influenced by diet, environment, and the microbiome.

**Aims:**

The purpose of this clinical study is to assess the safety and effects of a prebiotic‐containing gel cream on the skin microbiome of individuals with non‐cystic acne‐prone skin.

**Patients/Methods:**

In this 7‐week clinical trial, 30 eligible participants were recruited and enrolled from the Sacramento region. The study consisted of three visits: (1) screening; (2) week 0, baseline; (3) week 6. All participants received a standard non‐comedogenic cleanser and a prebiotic‐containing gel cream. The primary endpoint in this study was alteration in *C*. *acnes* abundance in mild to moderate non‐cystic, acne‐prone skin. The secondary endpoint in this study was functional gene analysis for skin barrier and skin inflammation‐related genes from whole genome sequencing of the skin microbiome.

**Results:**

Acne lesions significantly reduced in non‐inflammatory lesions, inflammatory lesions, and total lesions after treatment (−36.0%, −34.5%, and −35.9%, respectively). On the glabella, there was a 12.5 log2 fold increase in abundance of a healthy strain of *C. acnes* that is not associated with acne vulgaris. Furthermore, there was a wide range of functional bacterial genetic changes that may be associated with increased collagen and glutathione production. The gel cream was rated very well tolerated, and there were no adverse effects reported.

**Conclusions:**

This prebiotic‐containing gel cream can be an effective form of management for acne vulgaris by creating beneficial shifts in the skin microbiome.

## 1 Introduction

Acne vulgaris is a common skin condition that affects over 85% of the population. It most commonly affects adolescents and young adults but can affect individuals of any age [1]. It is a chronic inflammatory condition affecting the pilosebaceous unit characterized by papules, pustules, or nodules that typically form on the face, upper extremities, or upper back [2]. The pathogenesis of acne is multifactorial and includes hypersensitivity of the sebaceous glands to androgens resulting in increased sebum production, damage to the skin barrier, hyperkeratinization, and inflammation leading to bacterial colonization of the follicles [3, 4].

The bacteria largely implicated in acne is *Cutibacterium acnes* (formerly known as *Propionibacterium acnes* ) which resides on the skin and pilosebaceous units [5]. *C. acnes* is a gram‐positive, facultative, anaerobic bacilli, which is a part of normal skin flora along with *C. avidum* , *C. granulosum* , and *C. humerusii* [6]. Treatment options for acne vulgaris include topical therapies, systemic antibiotics, hormonal agents, and oral isotretinoin [7, 8].

Functional genomics refers to the mechanism by which specific genes contribute to the health and disease of our body. Previous studies in acne have demonstrated specific strains of *C. acnes* that are implicated in acne formation. Ribotyping refers to the sequencing of the 16 s ribosomal RNA gene of the bacterial strains of *C. acnes*. On average, individuals harbor 3 ± 2 strains of *C. acnes* ribotypes (RT) with RT's 4,5,7,8,9, and 10 found predominantly in acne patients. Additionally, RT's 1,2, and 3 can be found in all patients, while RT6 is associated with normal skin [9].

The bacteria's unique traits contribute to its pathogenic nature. It has been proven to increase seborrhea, increase inflammation, and cause hyperkeratinization of the pilosebaceous unit. It forms a biofilm, which allows it to adhere to the sebum and leads to the formation of microcomedones [6]. Additionally, the biofilm allows for macrophage evasion and avoidance of phagocytosis [10, 11].

Imbalance of microorganisms on the skin can lead to skin disorders. Probiotics, which can modulate the microbiome, are live microorganisms that are traditionally taken as an oral supplement to support gut and skin health [8]. Prebiotics are substances that are fermented by bacteria and allow for the proliferation of the microbiome. Inulin and fructooligosaccharide (FOS) are prebiotics that in recent years have been used as topical agents in treating skin conditions such as acne, rosacea, and psoriasis [8]. While there is limited data on the efficacy of the topical application of prebiotics, it is suggested that prebiotics can combat these conditions by repairing the skin barrier and reducing inflammation [12].

Squalane is the result of squalene hydrogenation and is found in sebum. Squalane oil can readily penetrate human skin and can increase the absorption of other active ingredients [13]. *Garcinia indica* , also known as kokum, is a tropical tree plant originating in India. Studies have shown the pharmaceutical effects of the plant, including its antimicrobial, anti‐inflammatory, and antioxidant properties [14].

In this study, we compare the effects of a prebiotic‐containing gel cream on skin microbiota and the degree of acne improvement in participants with mild to moderate non‐cystic acne. This intervention contains inulin, FOS, wax from seeds of *Garcinia indica* , and squalane as its key ingredients.

## 2 Materials and Methods

### 2.1 Subjects

This clinical study was conducted from September 2023 to January 2024 at Integrative Skin Science and Research in Sacramento, California. The Allendale Institutional Review Board approved the study on August 29, 2023, and the study was listed on clinicaltrials.gov (NCT05941065). Participants were recruited from the greater Sacramento area, and all participants provided written informed consent prior to enrollment.

### 2.2 Inclusion and Exclusion Criteria

The inclusion criteria for the study were as follows: men and women aged 18–35, men willing to shave their facial hair regularly (approximately every three days) for the study duration, presence of mild to moderate non‐cystic acne based on investigator global assessment (at least 5 inflammatory lesions and 10 non‐inflammatory lesions), all skin types (normal, oily, etc.), and all Fitzpatrick skin types (I–VI). Participants also agreed not to introduce any new colored cosmetics (lipsticks, eye shadows, facial foundations, blush, powder) on their face, to avoid sun exposure or use non‐comedogenic and non‐acnegenic sunscreen if sun exposure was unavoidable, and to refrain from professional or facial spa procedures for the study duration.

The exclusion criteria for the study were as follows: individuals who are pregnant or breastfeeding, prisoners, those unable to provide consent, those with severe or cystic acne, those unwilling to discontinue all facial topical products except the cleanser and the product provided in the study, those who were exposed to oral antibiotics within the previous month, those who had changed their hormonal contraception within three months prior to joining the study, current tobacco smokers or individuals with a smoking history of over 10 pack‐years, those who had started hormone replacement therapies (HRT) or hormones for birth control less than three months prior to the study or plan to start, stop, or change doses of HRT or hormones for birth control during the study. Additionally, individuals who had not taken any prescription (oral or topical) antibiotics, inhaled steroids (except for allergies), or hormones (pre‐ or post‐menopausal hormone‐replacement therapy, insulin, etc.), or other medications that were determined to make the skin more sensitive or affect the skin within one month were excluded. Those who had taken any prescription topical medication for acne in the two weeks prior to the study, used any topical prescription retinoids, azelaic acid, benzoyl peroxide, dapsone, sodium sulfacetamide, or similar prescription drugs on the face within one month prior to Visit 1, were on Accutane or other oral retinoids within six months prior to visit 1, or used light therapy or OTC topical medications/products (including anti‐acne or antibacterial agents, topical anti‐inflammatories, topical retinoids, etc.) on the face within two weeks prior to visit 1, were also excluded.

### 2.3 Investigational Products

All participants received a Cetaphil cleanser (Galderma Laboratories, Fort Worth, TX) and prebiotic‐containing gel cream (Study Sponsor). The ingredients of the gel cream included: water, caprylic/capric triglyceride, glycerin, *garcinia indica* seed butter, inulin, squalane, polyglyceryl‐6 distearate, FOS, propanediol, sclerotium gum, *beta vulgaris* (beet) root extract, jojoba esters, silica, sodium PCA, polyglyceryl‐3 beeswax, ethyl lauroyl arginate HCl, cetyl alcohol, potassium lactate, potassium sorbate, lactic acid, sodium phytate, xanthan gum, tocopherol, citric acid, lecithin, *glycine soja* (soybean) oil, pullulan, alcohol, and phenoxyethanol.

Participants were instructed to use a dime‐sized amount of the cleanser, applying it to their face in gentle circular motions and rinsing with cold or lukewarm water twice daily. Following the use of the cleanser, they were to apply a dime‐sized (~1 mL) amount of prebiotic‐containing gel cream to their face until it was gently absorbed, also twice daily. Additionally, participants were given a product log to document their daily usage of both products until their follow‐up visit.

### 2.4 Study Visits and Procedures

Written informed consent was obtained prior to enrollment. The study consisted of three visits: (1) screening, (2) week 0 (baseline), and (3) week 6. At the screening visit, if no washout was needed, participants began a one‐week wash‐in period using the cleanser exclusively before the baseline visit. At baseline, participants started using the Sponsor‐provided gel cream. Facial photography was obtained and analyzed at baseline and week 6 using the BTBP 3D Clarity Pro Facial Modeling and Analysis System (Brigh‐Tex BioPhotonics, San Jose, CA, USA). A Sebumeter (Delfin Technologies, Kuopio, Finland) was used on the left cheek and glabella at baseline and week 6 to assess sebum production. Skin microbiome swab samples were collected from the right cheek and glabella at both visits using skin swabs. At week 6, participants completed a tolerability questionnaire to assess how well the product was tolerated, rated on a 4‐point scale (0–3): 0: none, no itching; 1: mild, slight itching, not really bothersome; 2: moderate, definite itching that is somewhat bothersome; 3: severe, intense itching that may interrupt daily activities and/or sleep. All collected measurements and images were de‐identified and coded.

### 2.5 Acne Grading

Baseline and week 6 acne lesion counts were completed by two trained investigators, and the results were averaged. Graders assessed the number of non‐inflammatory lesions (NIL), inflammatory lesions (IL), total lesions (TL), and investigator global assessment (IGA).

### 2.6 Microbiome Analysis

#### 2.6.1 DNA Extraction and Quantification

DNA was isolated using the Zymo MicroPrep Kit and quantified using the Qubit Flex fluorometer and QubitTM dsDNA HS Assay Kit (Thermofisher Scientific).

#### 2.6.2 Library Preparation and Sequencing Methods

DNA libraries were prepared using the Watchmaker DNA Library Prep Kit and compatible Twist Universal Adapter System. After the genomic DNA was fragmented with a mastermix of Watchmaker Frag/AT Buffer and Frag/AT Enzyme Mix, 16 cycles of PCR were completed. Purification of DNA libraries was completed by AMPure magnetic beads (Beckmman Coulter) and eluted in nuclease‐free water. They were quantified using the QubitTM fluorometer dsDNA HS Assay Kit and sequenced on the Element AVITI platform using the AVITI 2 × 150 Cloudbreak sequencing kit.

#### 2.6.3 Taxonomic Analysis

The output of the pre‐computational phase of the k‐mer algorithm is a phylogeny tree of microbes. Results return taxonomic and relative abundance estimates for the microbial NGS datasets. The results were filtered using a threshold derived from internal statistical scores.

#### 2.6.4 Functional Analysis

Quality control and preprocessing of the sequencing output were completed with Bbduck [15]. The reads were then searched among the UniRef 90 database [16]. Reads were associated with gene sequences based on mapping quality, coverage, and sequence length, following the methods of previous literature [17]. Genes were then allocated to MetaCyc [18] reactions (Metabolic Enzymes) and GO terms [19].

### 2.7 Statistical Analysis

To compare baseline measurements to week 6 measurements, two‐tailed Student's t‐tests were used. Statistical significance was defined as *p* ≤ 0.05. Results were presented as mean and standard error of the mean. Data collected at week 0 served as the control for comparisons with week 6 data. Data visualization was performed using Prism v. 10 (GraphPad Software LLC, San Diego, CA, USA).

## 3 Results

**FIGURE 1:**
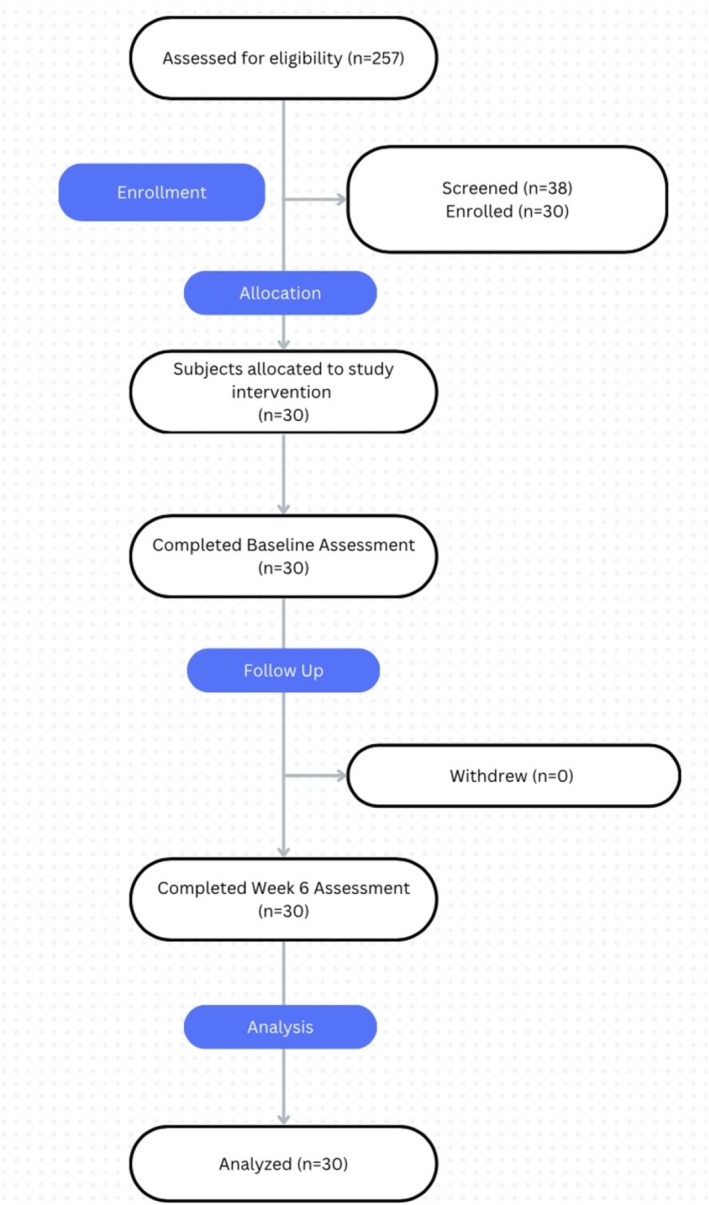
CONSORT Diagram of enrolled participants.

Of the total 38 subjects screened, 30 subjects met all inclusion/exclusion criteria and were enrolled (Figure 1). The average age of enrolled subjects was 25 ± 7 years and the cohort consisted of 29 females (97%) and 1 male (3%). Fitzpatrick skin types (FST) of the cohort included FST 1 (1), FST 2 (6), FST 3 (8), FST 4 (12), and FST 5 (3). There were no dropouts in this study.

### 3.1 Acne Lesion Grading

**FIGURE 2:**
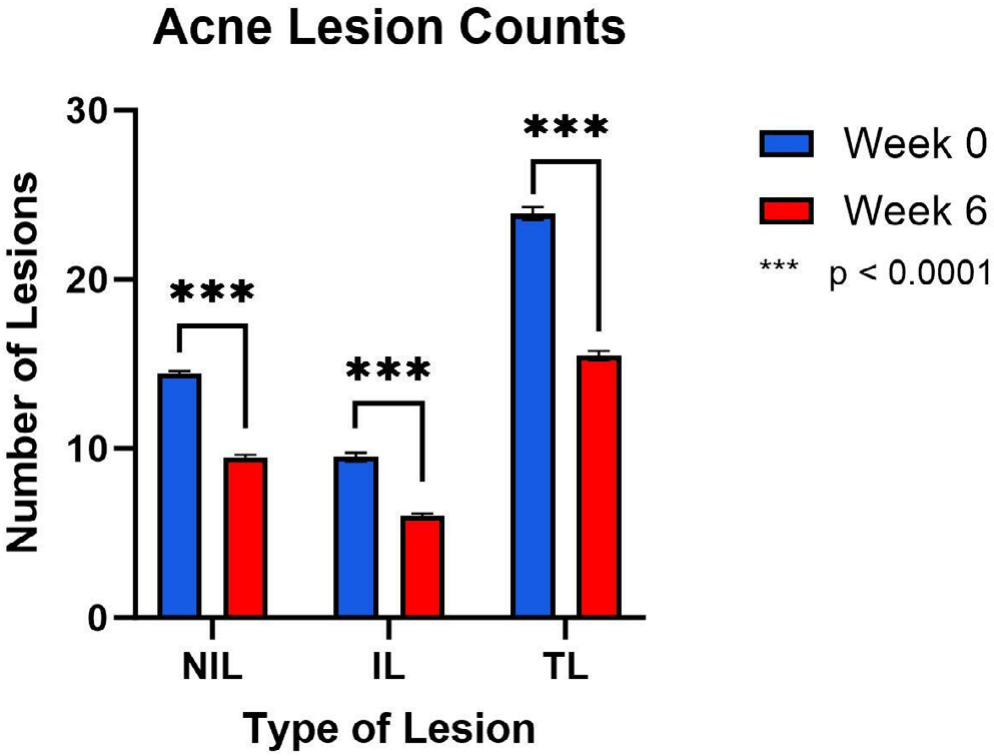
Following 6 weeks of topical application, there was a significant reduction in non‐inflammatory lesions (NIL), inflammatory lesions (IL), and total lesions (TL).

The baseline averages of acne lesion counts were 14.4 (± 0.2) NIL, 9.5 (±0.3) IL, and 23.9 (±0.4) TL. The median baseline IGA was 3. Following 6 weeks of treatment, NIL reduced by 36.0% (*p* < 0.0001), IL reduced by 34.5% (*p* < 0.0001), and TL reduced by 35.9% (*p* < 0.0001) (Figure 2).

### 3.2 Sebumeter

There were no statistically significant changes in sebum production after the treatment period. Sebum production in the left cheek increased by 59% (*p* = 0.07) and the change in the glabella was noted to be 6.7% and non‐significant (*p* = 0.6).

### 3.3 Skin Microbiome Abundance

A large variety of species had significant changes in abundance on the glabella and cheek (Tables S6 and S7). On the glabella, *C. acnes* [HL050PA2] had a log2 fold increase of 12.5 (*p* < 0.0001), *Gardnerella* [DNF01162] increased by 4.8 (*p* < 0.0001), and *C. durum* [F0235] increased by 4.7 (*p* < 0.001). The glabella also had species that were significantly reduced in abundance, such as *Cutibacterium* [sp. 5 U 42AFAA] by a log2 fold change of −9.8 (*p* < 0.0001), *Kocuria* [sp. HMSC066H03] by −5.9 (*p* < 0.0001), and *G. haemolysans* [ATCC 10379] by −5.4 (*p* < 0.0001).

On the left cheek, the three strains that increased in abundance the most include *C. tuberculostearicum* [SK141] with a log2 fold increase of 6.4 (*p* < 0.0001), *C. pseudogenitalium* [ATCC 33035] 6.4 (*p* < 0.0001), and *Staphylococcus* [sp. HMSC034A07] (*p* < 0.0001). While the following strains had the largest reduction: *Cutibacterium* [sp. 5 U 42AFAA] by −6.6 (*p* < 0.0001), *E. aerosaccus [*SK60] by −6.3 (*p* < 0.0001), and *P. rhinitis* [1–13] by −4.7 (*p* < 0.0001).

When assessing the overall changes in *C. acnes, Staphylococcus epidermidis*, and *Staphylococcus aureus* , there was a significant 856‐fold increase in the relative abundance (*p* < 0.001) of *S. epidermidis* (Table 1). However, when assessing for shifts at the level of the *Cutibacterium* genus (Table S1), *Cutibacterium acnes* HL050PA2 had a 5633‐fold increase in its relative abundance while all of the other *C. acnes* strains had no significant change. However, other *Cutibacterium* strains were noted to shift as *Cutibacterium namnetense* SK182B‐JCVI relative abundance decreased by 8.9‐fold (*p* < 0.001). When assessing for changes in the strains of *S. epidermidis* , the *S. epidermidis* VCU071 strain increased by 12.7‐fold (*p* < 0.001) whereas the other strains of *S. epidermidis* did not shift with the application of the prebiotic containing gel cream.

**TABLE 1:** Change in the relative abundance of main skin taxa after twice daily treatment with prebiotic‐containing gel cream for 6 weeks.

Taxon	Log2 fold change	Absolute change	*p*
Cutibacterium_acnes	−0.5993	−1.5149	0.6500
Staphylococcus_aureus	1.7127	3.2777	0.0691
Staphylococcus_epidermidis	9.7421	856.3736	3.60E−26

### 3.4 Functional Microbiome Data

There was a variety of functional MetaCyc pathways that were significantly upregulated or downregulated in both the glabella and cheek (Tables S8 and S9). Relevant pathways can be found in Table 2.

**TABLE 2:** Relevant MetaCyc pathways which had significant changes in expression following treatment.

Anatomical location	MetaCyc ID	Log2 fold change	Fold change	*p*
Left cheek	pyruvate_fermentation_to_acetate_and_S_lactate_I	−2.296	0.204	< 0.0001
	pyruvate_fermentation_to_acetate_and_lactate_II	−2.038	0.243	0.0030
	pyruvate_fermentation_to_isobutanol_engineered	−2.032	0.244	0.0243
	L_lysine_biosynthesis_II	3.857	14.486	< 0.0001
	L_ornithine_biosynthesis_II	4.998	31.964	< 0.0001
Glabella	L_ornithine_biosynthesis_II	−3.255	0.105	< 0.0001
	superpathway_of_L_cysteine_biosynthesis_mammalian	3.632	12.401	< 0.0001

*Note:* A full list of all significant pathways can be found in Tables S3 and S4.

### 3.5 Skin Tolerability

Based on the tolerability assessment questionnaire, we collected valuable data regarding the subjective experience of using the prebiotic containing gel cream (Table 3). Notably, 86% of subjects reported no itching, 93% of subjects reported no burning, and 93% of subjects reported no scaling or peeling. There were no adverse events reported in this study.

**TABLE 3:** Results from the tolerability assessment questionnaire.

Severity	Itching	Burning	Scaling/Peeling	Stinging
None	86%	93%	93%	100%
Mild	10%	7%	3%	0%
Moderate	3%	0%	3%	0%
Severe	0%	0%	0%	0%

*Note:* This questionnaire reports subjective feelings of itching, burning, scaling/peeling, and stinging.

## 4 Discussion

The prebiotic containing gel cream of this study demonstrated beneficial effects on acne lesion counts and in shifts of the skin microbiome. The key components of the gel cream are essential to producing its effects. Inulin and FOS are prebiotics that can stimulate the growth of bacteria within the skin microbiome [20]. This has likely initiated the various changes in the bacterial flora following the treatment phase.

Multiple bacterial strains had significant changes in abundance following the treatment period. *C. acnes* HL050PA2 had the highest (5633‐fold) increase in relative abundance. While certain strains of *C. acnes* have been shown to facilitate acne lesions via release of IL‐1β [21], this strain, belonging to phylotype 2, is associated with healthy non‐acne prone skin [22]. Furthermore, *S. epidermidis* increased 856‐fold while the *S. epidermidis* VCU071 strain increased by 12.7‐fold. Research has shown that *S. epidermidis* can reduce the abundance of pathogenic strains of *C. acnes* [23]. The bacteria in this study were fermented with glycerol, which is an endogenous substance produced by our skin and is included in many modern skin products [21]. Notably, there were no significant changes in the relative abundance of *C. acnes* when measured from the cheek. Our findings reveal that descriptions of the skin microbiota need to be more nuanced with greater discussion of the particular strains. Here, we found that no strains of *C. acnes* decreased and that one of the healthy strains increased instead. In fact, *C. namenetense* was decreased after moisturizer use, raising the possibility that other taxa within the *Cutibacterium* genus may be relevant for acne and that the focus on *C. acnes* may be too restrictive.

Not only did the prebiotic containing gel cream modulate the abundance of the microbiome, but it importantly induced changes in microbial gene expression. In the cheek, the expression of the L‐ornithine biosynthesis II pathway increased by 32‐fold and L‐lysine biosynthesis II increased by 14.5‐fold. Ornithine acts as a precursor to proline [24] which, along with lysine, consists of some of the building blocks of collagen [25]. In fact, one of the major rate‐limiting steps of collagen biosynthesis involves the isomerization of proline residues [26]. As the major component of the extracellular skin matrix, collagen can improve skin elasticity, firmness, and skin hydration [27].

Furthermore, on the cheek, three pathways of pyruvate fermentation were reduced. Pyruvate has been shown to reduce oxidative stress by reducing mitochondrial reactive oxygen species generation and stabilizing the mitochondrial membrane potential during oxidative stress [28], thus providing evidence of pyruvate's potential to ameliorate skin inflammation and ultraviolet (UV) damage. These findings are supported by trials that have demonstrated that pyruvic acid peels can improve acne, reduce hyperpigmentation, increase skin elasticity, and improve signs of photoaging [29, 30, 31]. Furthermore, exogenous pyruvate has demonstrated in vitro improvement of hyperpigmentation [32].

The glabella demonstrated differing functional microbiome changes. The pathway of production of L‐cysteine was upregulated by 12‐fold. Cysteine is a key component of glutathione production, and its bioavailability is one of the key regulators of the rate‐limiting step of glutathione biosynthesis [33, 34]. Glutathione supplementation has demonstrated improvement in hyperpigmentation [35] and wrinkles [36]; cysteine's contribution to glutathione production can be validated by research demonstrating that cysteine supplementation can improve skin brightening [37].

Interestingly, the glabella demonstrated opposite effects of the cheek on L‐ornithine with a 90% downregulation of the L‐ornithine biosynthesis II pathway. It is unclear if the variability of application or the biophysical characteristics between the two anatomical areas had a significant impact on the differences in bacterial proliferation and bacterial function that we discovered.

The varying properties of the stratum corneum throughout the face may influence the shifts we found in the microbiome of the cheek and glabella. Previous research has demonstrated different functional measurements in different locations of the face; for example, the cheek has demonstrated less TEWL than the nasolabial fold, chin, tip of the nose, or forehead [38]. Additionally, uneven topical application can lead to variations in regional results.

Our research agrees with previous literature which finds that topical prebiotics can assist in shifts of the microbiome. An in vitro study found that topical prebiotic konjac glucomannan hydrolysates significantly enhanced the probiotic inhibition of *Propionibacterium acnes* NCTC 737 [39]. However, more human clinical trials are needed to explore further the results in humans.

There were several limitations to this study. The study was relatively small, but each person was used as their own control, and there were no dropouts. Nevertheless, the results shown here warrant a large skin microbiome‐focused study in the future. This study globally looked at acne, but the skin microbiome shifts were not differentiated between inflammatory and non‐inflammatory lesions.

## 5 Conclusion

With minimal issues of skin tolerability and significant reductions in acne lesion count, the prebiotic containing gel cream shows promise in the management of acne vulgaris. The prebiotic formulation of this intervention may have induced shifts in the skin bacterial microbiome into a state that is less associated with acne‐prone skin. This was associated with changes in the functional genetic composition of this bacteria consistent with improved collagen and glutathione production.

## Author Contributions

Study Conception and Design (Laila Afzal, Nasima Afzal, Hemali B. Gunt, Raja K. Sivamani), Data Collection (Laila Afzal, Nhi Nguyen, Nasima Afzal), Analysis and Interpretation of Results (Ajay S. Dulai, Zill‐e‐huma Khan), Draft Manuscript Preparation (Laila Afzal, Ajay S. Dulai, Zill‐e‐huma Khan, Hemali B. Gunt, Raja K. Sivamani).

## Ethics Statement

This study was approved by the Allendale IRB, and all participants provided written informed consent prior to participation.

## Conflicts of Interest

Raja K. Sivamani serves as a scientific advisor for LearnHealth, Codex Labs, and Arbonne and as a consultant to Burt's Bees, Novozymes, Nutrafol, Abbvie, Leo, Almirall, Galderma, Lilly, UCB, Incyte, Sanofi, Novartis, Sun and Regeneron Pharmaceuticals. The other authors declare no conflicts of interest.

## Supporting information


Table S1.

Table S2.

Table S3.

Table S4.

Table S5.

Table S6.

Table S7.

Table S8.

Table S9.

## Data Availability

The data is not publicly available. The data presented in this study are available on reasonable request from the corresponding author.
