# Supplementary material for: Open‐Label, Prospective Study of a Prebiotic Gel Cream on Its Efficacy of Mild to Moderate Acne Management and Effects on the Functional Skin Microbiome
Source: J Cosmet Dermatol. 2025 Oct 16;24(10):e70138. doi: 10.1111/jocd.70138 (PMC12529085; doi:10.1111/jocd.70138)
Supplement: Supplementary file 1 — Table S1. [file JOCD-24-e70138-s006.docx]

Supplementary 1 – Change in the relative abundance of *Cutibacterium* strains after twice daily treatment with prebiotic containing gel cream for 6 weeks

| **Taxon (Strain)** | **Log2 Fold Change** | **Absolute Change** | **P Value** |
| --- | --- | --- | --- |
| Cutibacterium_acnes_hdn-1 | 0.0017 | 1.0012 | 0.9969 |
| Cutibacterium_acnes_HL025PA1 | 0.0584 | 1.0413 | 0.8951 |
| Cutibacterium_acnes_HL027PA1 | 0.0584 | 1.0413 | 0.8951 |
| Cutibacterium_acnes_HL027PA2 | -0.0551 | -1.0389 | 0.9011 |
| Cutibacterium_acnes_HL030PA2 | -0.0551 | -1.0389 | 0.9009 |
| Cutibacterium_acnes_HL050PA2 | 12.4598 | 5633.3323 | 0.0000 |
| Cutibacterium_acnes_J139 | -0.0550 | -1.0388 | 0.9012 |
| Cutibacterium_acnes_KPA171202 | 0.0016 | 1.0011 | 0.9971 |
| Cutibacterium_granulosum | -1.7639 | -3.3961 | 0.1350 |
| Cutibacterium_granulosum_DSM_20700 | 0.9822 | 1.9754 | 0.3792 |
| Cutibacterium_granulosum_TM11 | 0.0584 | 1.0413 | 0.8952 |
| Cutibacterium_namnetense_SK182B-JCVI | -3.1514 | -8.8851 | 1.07E-04 |
| Cutibacterium_sp_5_U_42AFAA | -9.7918 | -886.3927 | 1.32E-24 |
| Cutibacterium_sp_HMSC065F07 | 0.2086 | 1.1555 | 0.8740 |
| Cutibacteirum_sp_KPL1844 | 0.5195 | 1.4335 | 0.6050 |
