# Supplementary material for: Open‐Label, Prospective Study of a Prebiotic Gel Cream on Its Efficacy of Mild to Moderate Acne Management and Effects on the Functional Skin Microbiome
Source: J Cosmet Dermatol. 2025 Oct 16;24(10):e70138. doi: 10.1111/jocd.70138 (PMC12529085; doi:10.1111/jocd.70138)
Supplement: Supplementary file 2 — Table S2. [file JOCD-24-e70138-s009.docx]

Supplementary 2 – Change in the relative abundance of *Staphylococcus epidermidis* strains after twice daily treatment with prebiotic containing gel cream for 6 weeks

| **Taxon (Strain)** | **Log2 Fold Change** | **Absolute Change** | **P Value** |
| --- | --- | --- | --- |
| Staphylococcus_epidermidis_AG42 | -1.5152 | -2.8584 | 0.1173 |
| Staphylococcus_epidermidis_BCM-HMP0060 | 0.0017 | 1.0012 | 0.9969 |
| Staphylococcus_epidermidis_M23864_W2_grey | 0.0017 | 1.0012 | 0.9969 |
| Staphylococcus_epidermidis_NIHLM001 | 0.1174 | 1.0848 | 0.7929 |
| Staphylococcus_epidermidis_NIHLM003 | 0.0017 | 1.0012 | 0.9969 |
| Staphylococcus_epidermidis_NIHLM008 | 0.0017 | 1.0012 | 0.9969 |
| Staphylococcus_epidermidis_NIHLM039 | 0.0584 | 1.0413 | 0.8951 |
| Staphylococcus_epidermidis_PM221 | 0.0585 | 1.0414 | 0.8949 |
| Staphylococcus_epidermidis_SK135 | 0.4076 | 1.3264 | 0.7233 |
| Staphylococcus_epidermidis_VCU071 | 3.6655 | 12.6886 | 5.05E-08 |
