# Supplementary material for: Open‐Label, Prospective Study of a Prebiotic Gel Cream on Its Efficacy of Mild to Moderate Acne Management and Effects on the Functional Skin Microbiome
Source: J Cosmet Dermatol. 2025 Oct 16;24(10):e70138. doi: 10.1111/jocd.70138 (PMC12529085; doi:10.1111/jocd.70138)
Supplement: Supplementary file 4 — Table S4. [file JOCD-24-e70138-s004.docx]

**Supplementary Table 7:** Significant changes in strains measured at the left cheek

| Strain | log2 Fold Change | Fold Change | p value |
| --- | --- | --- | --- |
| Moraxella_osloensis | -6.945 | -123.215 | <0.0001 |
| Propionibacterium_sp_5_U_42AFAA | -6.600 | -96.985 | <0.0001 |
| Enhydrobacter_aerosaccus_SK60 | -6.297 | -78.644 | <0.0001 |
| Peptoniphilus_rhinitidis_1-13 | -4.677 | -25.575 | <0.0001 |
| Neisseria_sp_HMSC072F04 | -4.347 | -20.348 | <0.0001 |
| Abiotrophia_sp_HMSC24B09 | -3.610 | -12.214 | <0.0001 |
| Peptoniphilus_lacydonensis | -3.438 | -10.837 | <0.0001 |
| Actinomyces_naeslundii_str_Howell_279 | -3.252 | -9.529 | <0.0001 |
| Lautropia_mirabilis_ATCC_51599 | -3.079 | -8.448 | <0.0001 |
| Propionibacterium_namnetense_SK182B-JCVI | -3.049 | -8.276 | <0.0001 |
| Actinobaculum_sp_oral_taxon_183_str_F0552 | -2.967 | -7.820 | <0.0001 |
| Neisseria_u_t | -2.734 | -6.653 | <0.0001 |
| Porphyromonadaceae_bacterium_KA00676 | -2.728 | -6.626 | <0.0001 |
| Enhydrobacter_sp_H5 | -2.452 | -5.472 | 0.0042 |
| Pyrinomonas_methylaliphatogenes | -2.399 | -5.273 | <0.0001 |
| Dialister_invisus_DSM_15470 | -2.343 | -5.075 | <0.0001 |
| Gemella_morbillorum_M424 | -2.294 | -4.906 | <0.0001 |
| Bradyrhizobium_sp_MOS004 | -1.999 | -3.997 | <0.0001 |
| Meiothermus_silvanus_DSM_9946 | -1.571 | -2.972 | 0.0195 |
| Staphylococcus_epidermidis_VCU071 | 1.899 | 3.731 | 0.0413 |
| Corynebacterium_matruchotii | 1.917 | 3.776 | 0.0226 |
| Propionibacteriaceae_u_t | 1.942 | 3.842 | 0.0111 |
| Staphylococcus_aureus | 1.967 | 3.909 | 0.0148 |
| Staphylococcus_u_t | 2.130 | 4.377 | 0.0018 |
| Corynebacterium_pseudodiphtheriticum_090104 | 2.186 | 4.549 | <0.0001 |
| Anaerococcus_nagyae | 2.189 | 4.559 | <0.0001 |
| Corynebacterium_matruchotii_ATCC_14266 | 2.236 | 4.712 | <0.0001 |
| Staphylococcus_capitis_CR01 | 2.279 | 4.854 | <0.0001 |
| Streptococcus_sp_M334 | 2.460 | 5.503 | <0.0001 |
| Janibacter_hoylei_PVAS-1 | 2.612 | 6.113 | <0.0001 |
| Staphylococcus_capitis_C87 | 2.684 | 6.427 | 0.0051 |
| Actinomyces_sp_ICM39 | 2.696 | 6.481 | <0.0001 |
| Neisseria_sicca_VK64 | 2.760 | 6.775 | <0.0001 |
| Corynebacterium_propinquum_DSM_44285 | 2.948 | 7.715 | <0.0001 |
| Haemophilus_sp_HMSC068C11 | 3.161 | 8.945 | <0.0001 |
| Veillonella_sp_oral_taxon_158_str_F0412 | 3.368 | 10.326 | <0.0001 |
| Dolosigranulum_pigrum_ATCC_51524 | 3.394 | 10.513 | <0.0001 |
| Paracoccus_yeei_ATCC_BAA-599 | 3.416 | 10.671 | <0.0001 |
| Corynebacterium_sp_KPL1824 | 3.485 | 11.197 | <0.0001 |
| Pseudomonas_u_t | 3.509 | 11.385 | <0.0001 |
| Xanthomonas_campestris_pv_campestris_str_ATCC_33913 | 3.578 | 11.940 | <0.0001 |
| Paracoccus_aeridis | 3.670 | 12.730 | <0.0001 |
| Micrococcus_aloeverae | 4.325 | 20.043 | <0.0001 |
| Gemella_haemolysans_ATCC_10379 | 4.345 | 20.318 | <0.0001 |
| Lactobacillus_crispatus | 4.434 | 21.621 | <0.0001 |
| Acinetobacter_u_t | 4.462 | 22.042 | <0.0001 |
| Xanthomonas_campestris_pv_campestris | 4.571 | 23.763 | <0.0001 |
| Staphylococcus_epidermidis_NIHLM039 | 5.164 | 35.847 | <0.0001 |
| Staphylococcus_sp_HMSC034A07 | 5.439 | 43.377 | <0.0001 |
| Prevotella_bivia | 5.673 | 51.029 | <0.0001 |
| Corynebacterium_pseudogenitalium_ATCC_33035 | 6.401 | 84.514 | <0.0001 |
| Corynebacterium_tuberculostearicum_SK141 | 6.449 | 87.346 | <0.0001 |
