# Supplementary material for: Open‐Label, Prospective Study of a Prebiotic Gel Cream on Its Efficacy of Mild to Moderate Acne Management and Effects on the Functional Skin Microbiome
Source: J Cosmet Dermatol. 2025 Oct 16;24(10):e70138. doi: 10.1111/jocd.70138 (PMC12529085; doi:10.1111/jocd.70138)
Supplement: Supplementary file 5 — Table S5. [file JOCD-24-e70138-s001.docx]

**Supplementary Table 8:** Functional changes in MetaCyc pathways measured at the glabella

| MetaCyc ID | log2 Fold Change | Fold Change | p value |
| --- | --- | --- | --- |
| heme_b_biosynthesis_V_aerobic | -6.209 | -73.962 | <0.0001 |
| phosphatidylglycerol_biosynthesis_I_plastidic | -5.144 | -35.367 | <0.0001 |
| phosphatidylglycerol_biosynthesis_II_non_plastidic | -5.103 | -34.378 | <0.0001 |
| thiamine_diphosphate_salvage_IV_yeast | -4.997 | -31.933 | <0.0001 |
| glucose_and_glucose_1_phosphate_degradation | -4.289 | -19.551 | <0.0001 |
| superpathway_of_UDP_gluco_ding_blocks_biosynthesis | -4.285 | -19.494 | <0.0001 |
| UDP_N_acetyl_D_glucosamine_biosynthesis_II | -4.152 | -17.776 | <0.0001 |
| CDP_diacylglycerol_biosynthesis_I | -3.952 | -15.475 | <0.0001 |
| CDP_diacylglycerol_biosynthesis_II | -3.860 | -14.524 | <0.0001 |
| superpathway_of_phospholi_biosynthesis_I_bacteria | -3.625 | -12.337 | <0.0001 |
| L_ornithine_biosynthesis_II | -3.255 | -9.546 | <0.0001 |
| ethanolamine_utilization | -3.139 | -8.812 | <0.0001 |
| acetylene_degradation_anaerobic | -2.865 | -7.285 | <0.0001 |
| glycogen_degradation_I | -2.444 | -5.441 | <0.0001 |
| ketogenesis | -2.268 | -4.818 | <0.0001 |
| 4_amino_2_methyl_5_diphos_rimidine_biosynthesis_II | -2.199 | -4.592 | <0.0001 |
| L_citrulline_biosynthesis | -2.185 | -4.547 | 0.0305 |
| colanic_acid_building_blocks_biosynthesis | -2.126 | -4.364 | <0.0001 |
| superpathway_of_L_alanine_biosynthesis | -2.120 | -4.347 | <0.0001 |
| C4_photosynthetic_carbon__lation_cycle_NAD_ME_type | -2.022 | -4.061 | <0.0001 |
| Entner_Doudoroff_pathway_I | -1.949 | -3.861 | <0.0001 |
| 8_amino_7_oxononanoate_biosynthesis_I | -1.911 | -3.761 | <0.0001 |
| superpathway_of_L_phenylalanine_biosynthesis | -1.859 | -3.628 | <0.0001 |
| biotin_biosynthesis_I | -1.847 | -3.597 | <0.0001 |
| superpathway_of_L_methion_thesis_transsulfuration | -1.814 | -3.516 | 0.0011 |
| urea_cycle | -1.779 | -3.432 | 0.0013 |
| superpathway_of_L_homoser__methionine_biosynthesis | -1.774 | -3.419 | 0.0013 |
| superpathway_of_S_adenosyl_L_methionine_biosynthesis | -1.766 | -3.401 | 0.0014 |
| tRNA_processing | -1.539 | -2.905 | 0.0042 |
| NAD_de_novo_biosynthesis_I_from_aspartate | -1.384 | -2.610 | 0.0079 |
| adenosine_nucleotides_degradation_II | -1.317 | -2.491 | 0.0113 |
| lipid_IVA_biosynthesis_E__coli | -1.228 | -2.343 | 0.0160 |
| lipid_IVA_biosynthesis_P__putida | -1.228 | -2.343 | 0.0160 |
| C4_photosynthetic_carbon__ation_cycle_NADP_ME_type | -1.184 | -2.272 | 0.0204 |
| guanosine_nucleotides_degradation_III | -1.134 | -2.195 | 0.0251 |
| L_N_delta_acetylornithine_biosynthesis | -1.054 | -2.076 | 0.0353 |
| guanosine_nucleotides_degradation_II | -1.053 | -2.075 | 0.0352 |
| CMP_3_deoxy_D_manno_octulosonate_biosynthesis | -1.044 | -2.062 | 0.0368 |
| phosphatidylcholine_acyl_editing | -0.981 | -1.973 | 0.0478 |
| anaerobic_energy_metabolism_invertebrates_cytosol | 1.709 | 3.269 | 0.0477 |
| lactose_and_galactose_degradation_I | 1.746 | 3.354 | 0.0395 |
| sucrose_degradation_III_sucrose_invertase | 1.789 | 3.457 | 0.0298 |
| assimilatory_sulfate_reduction_I | 1.935 | 3.823 | 0.0487 |
| formaldehyde_oxidation_I | 2.115 | 4.333 | <0.0001 |
| assimilatory_sulfate_reduction_IV | 2.355 | 5.116 | 0.0087 |
| terminal_O_glycans_residu__precursor_disaccharide | 2.521 | 5.741 | <0.0001 |
| incomplete_reductive_TCA_cycle | 2.564 | 5.915 | <0.0001 |
| glyoxylate_cycle | 2.731 | 6.637 | <0.0001 |
| sucrose_degradation_IV_sucrose_phosphorylase | 2.781 | 6.876 | 0.0035 |
| O_antigen_building_blocks_biosynthesis_E__coli | 2.930 | 7.623 | <0.0001 |
| polyisoprenoid_biosynthesis_E__coli | 2.982 | 7.899 | <0.0001 |
| superpathway_of_L_cysteine_biosynthesis_mammalian | 3.632 | 12.401 | <0.0001 |
| superpathway_of_R_R_butanediol_biosynthesis | 3.766 | 13.603 | <0.0001 |
| superpathway_of_glucose_and_xylose_degradation | 3.796 | 13.893 | <0.0001 |
| all_trans_farnesol_biosynthesis | 4.028 | 16.309 | <0.0001 |
| superpathway_of_sulfur_am_accharomyces_cerevisiae | 4.099 | 17.131 | <0.0001 |
| phospholipases | 4.181 | 18.140 | <0.0001 |
| superpathway_of_N_acetylg_lneuraminate_degradation | 4.297 | 19.656 | <0.0001 |
| superpathway_of_L_methion_esis_by_sulfhydrylation | 4.550 | 23.431 | <0.0001 |
| gluconeogenesis_III | 5.015 | 32.334 | <0.0001 |
| octane_oxidation | 6.278 | 77.614 | <0.0001 |
| peptidoglycan_biosynthesis_II_staphylococci | 6.655 | 100.761 | <0.0001 |
| mixed_acid_fermentation | 7.193 | 146.293 | <0.0001 |
| superpathway_of_menaquinol_11_biosynthesis | 8.937 | 490.197 | <0.0001 |
| superpathway_of_menaquinol_12_biosynthesis | 8.937 | 490.197 | <0.0001 |
| superpathway_of_menaquinol_13_biosynthesis | 8.937 | 490.197 | <0.0001 |
| superpathway_of_menaquinol_7_biosynthesis | 8.971 | 501.683 | <0.0001 |
