# Supplementary material for: Open‐Label, Prospective Study of a Prebiotic Gel Cream on Its Efficacy of Mild to Moderate Acne Management and Effects on the Functional Skin Microbiome
Source: J Cosmet Dermatol. 2025 Oct 16;24(10):e70138. doi: 10.1111/jocd.70138 (PMC12529085; doi:10.1111/jocd.70138)
Supplement: Supplementary file 9 — Table S9. [file JOCD-24-e70138-s003.docx]

**Supplementary Table 9:** Significant changes in strains measured at the left cheek

| Strain | log2 Fold Change | Fold Change | p value |
| --- | --- | --- | --- |
| Propionibacterium_sp_5_U_42AFAA | -9.792 | -886.393 | <0.0001 |
| Pseudomonas_formosensis | -6.304 | -79.001 | <0.0001 |
| Kocuria_sp_HMSC066H03 | -5.911 | -60.182 | <0.0001 |
| Gemella_haemolysans_ATCC_10379 | -5.375 | -41.510 | <0.0001 |
| Streptococcus_sp_OH4692_COT-348 | -4.887 | -29.594 | <0.0001 |
| Micrococcus_sp_KT16 | -4.724 | -26.432 | <0.0001 |
| Sphingomonas_ginsenosidimutans | -4.656 | -25.216 | <0.0001 |
| Haemophilus_sputorum | -4.418 | -21.376 | <0.0001 |
| Roseomonas_mucosa_ATCC_BAA-692 | -4.101 | -17.165 | <0.0001 |
| Actinomyces_naeslundii_str_Howell_279 | -4.071 | -16.803 | <0.0001 |
| Porphyromonas_sp_oral_taxon_279_str_F0450 | -3.839 | -14.306 | <0.0001 |
| Streptococcus_oralis_SK100 | -3.683 | -12.847 | <0.0001 |
| Pseudomonas_sp_NBRC_111131 | -3.621 | -12.306 | <0.0001 |
| Streptococcus_sp_NPS_308 | -3.555 | -11.755 | <0.0001 |
| Corynebacterium_matruchotii | -3.439 | -10.844 | <0.0001 |
| Schaalia_odontolytica | -3.188 | -9.113 | <0.0001 |
| Pseudomonas_u_t | -3.155 | -8.907 | <0.0001 |
| Propionibacterium_namnetense_SK182B-JCVI | -3.151 | -8.885 | <0.0001 |
| Paenibacillus_sophorae_S27 | -3.108 | -8.623 | <0.0001 |
| Acinetobacter_johnsonii_SH046 | -2.987 | -7.931 | <0.0001 |
| Porphyromonadaceae_bacterium_KA00676 | -2.773 | -6.833 | <0.0001 |
| Brevibacterium_casei_S18 | -2.582 | -5.990 | <0.0001 |
| Prevotella_nanceiensis_DSM_19126_JCM_15639 | -2.063 | -4.179 | <0.0001 |
| Actinomyces_graevenitzii | -2.027 | -4.075 | <0.0001 |
| Prevotella_pallens_ATCC_700821 | -1.937 | -3.828 | <0.0001 |
| Methyloversatilis_universalis | -1.868 | -3.649 | 0.0077 |
| Sphingobium_limneticum | -1.639 | -3.115 | 0.0416 |
| Gemella_haemolysans_M341 | -1.393 | -2.626 | 0.0497 |
| Janibacter_hoylei_PVAS-1 | 1.857 | 3.622 | 0.0132 |
| Corynebacterium_lipophiloflavum_DSM_44291 | 1.969 | 3.915 | <0.0001 |
| Paracoccus_yeei_ATCC_BAA-599 | 2.034 | 4.095 | 0.0112 |
| Enterobacteriaceae_u_t | 2.110 | 4.316 | <0.0001 |
| Paracoccus_sp_MT-B08 | 2.116 | 4.336 | <0.0001 |
| Corynebacterium_tuberculostearicum_SK141 | 2.118 | 4.339 | 0.0225 |
| Corynebacterium_pseudogenitalium_ATCC_33035 | 2.153 | 4.447 | 0.0187 |
| Peptoniphilus_rhinitidis_1-13 | 2.211 | 4.629 | 0.0164 |
| Simonsiella_muelleri_ATCC_29453 | 2.263 | 4.801 | <0.0001 |
| Mycobacterium_gordonae | 2.416 | 5.337 | <0.0001 |
| Corynebacterium_sp_KPL1824 | 2.477 | 5.568 | 0.0028 |
| Streptococcus_thermophilus | 2.512 | 5.704 | <0.0001 |
| Kocuria_u_t | 2.515 | 5.718 | <0.0001 |
| Lactobacillales_u_t | 2.571 | 5.942 | <0.0001 |
| Corynebacterium_kroppenstedtii_DSM_44385 | 2.572 | 5.945 | <0.0001 |
| Campylobacter_ureolyticus | 2.598 | 6.056 | <0.0001 |
| Streptococcus_mitis | 2.737 | 6.666 | <0.0001 |
| Brachybacterium_muris_UCD-AY4 | 2.737 | 6.667 | <0.0001 |
| Brevibacterium_sp_CS2 | 2.816 | 7.044 | <0.0001 |
| Alloiococcus_otitis_ATCC_51267 | 2.828 | 7.103 | <0.0001 |
| Paracoccus_salipaludis | 2.849 | 7.205 | <0.0001 |
| Atopobium_vaginae_DSM_15829 | 2.901 | 7.469 | <0.0001 |
| Prevotella_bivia | 2.987 | 7.927 | <0.0001 |
| Sphingomonas_sp_1F27F7B | 3.016 | 8.092 | <0.0001 |
| Streptococcus_sp_HMSC062B01 | 3.396 | 10.529 | <0.0001 |
| Janibacter_indicus | 3.465 | 11.044 | <0.0001 |
| Corynebacterium_accolens_ATCC_49725 | 3.565 | 11.835 | <0.0001 |
| Staphylococcus_epidermidis_VCU071 | 3.665 | 12.689 | <0.0001 |
| Paracoccus_marinus | 3.668 | 12.715 | <0.0001 |
| Actinomyces_viscosus_C505 | 3.888 | 14.810 | <0.0001 |
| Williamsia_sp_D3 | 4.260 | 19.161 | <0.0001 |
| Williamsia_muralis_NBRC_105860 | 4.592 | 24.113 | <0.0001 |
| Gardnerella_vaginalis_5-1 | 4.664 | 25.359 | <0.0001 |
| Corynebacterium_durum_F0235 | 4.701 | 26.001 | <0.0001 |
| Paracoccus_mutanolyticus | 4.714 | 26.240 | <0.0001 |
| Gardnerella_sp_DNF01162 | 4.755 | 26.999 | <0.0001 |
| Staphylococcus_u_t | 5.519 | 45.858 | <0.0001 |
| Neisseria_u_t | 5.866 | 58.330 | <0.0001 |
| Paracoccus_aeridis | 6.523 | 91.951 | <0.0001 |
| Staphylococcus_epidermidis | 9.742 | 856.374 | <0.0001 |
| Cutibacterium_acnes_HL050PA2 | 12.460 | 5633.333 | <0.0001 |
